# A Case of Post-COVID-19 Rhino-Cerebral Mucormycosis in an Immunocompromised Patient

**DOI:** 10.7759/cureus.42652

**Published:** 2023-07-29

**Authors:** Labeeba Faria, Tasnova Mahin, Md Abdul Qader, Mosaddeque Ahmed, Muhammad A Anwar

**Affiliations:** 1 Nephrology, Square Hospitals Limited, Dhaka, BGD; 2 Internal Medicine, Square Hospitals Limited, Dhaka, BGD; 3 Pediatric Nephrology, Square Hospitals Limited, Dhaka, BGD

**Keywords:** posaconazole, liposomal amphotericin b, acute kideny injury, diabetes mellitus type 2, covid-19 infection, rhino-cerebral mucormycosis

## Abstract

Mucormycosis is a fulminant angioinvasive fungal infection that occurs in an immunocompromised condition, most commonly in diabetic patients. Rhino-cerebral and pulmonary infections are common but may also lead to disseminated disease associated with a high mortality rate (almost 100%). Here we report on an elderly diabetic lady presented with a headache and altered level of consciousness and peri-orbital swelling following coronavirus disease 2019 (COVID-19). Imaging studies revealed a single large space-occupying lesion in the frontal lobe extending to the left orbit and sinusitis. An excisional biopsy was taken from the middle meatus of the nasal cavity and histopathology findings were broad aseptate hyphae branching at the right angle which suggests mucormycosis. Liposomal amphotericin B was started but the patient developed acute kidney injury (AKI) and bi-cytopenia (thrombocytopenia and anemia) followed by sepsis resulting in death. Though this is a rapidly progressing disease with a high mortality rate, a strong level of suspicion and early diagnosis can save lives.

## Introduction

Mucormycosis, also known as zygomycosis, is a highly aggressive opportunistic infection caused by fungi belonging to the Mucoraceae family. It was initially described by Paultauf in 1885 and detected as the third most angioinvasive fungal infection, following candidiasis and aspergillosis [1,2]. Approximately 1.7 cases of mucormycosis are documented to occur per year for every 1,000,000 people [3]. In immunocompromised patients, the disease results from deteriorating immune system changes leading to rapid proliferation and invasion of the fungal organism into deeper tissues. Important predisposing factors for developing mucormycosis include lymphoma, leukemia, organ transplant, penetrating trauma, iron overload, and desferrioxamine therapy [4]. Notably, iron plays a significant role in the growth of mucormycosis, and an acidic state in the body leads to the release of more iron into the bloodstream [5].

Rhino-cerebral mucormycosis is the most prevalent form and typically starts with the inhalation of spores in the paranasal sinus. Tissue necrosis and black eschar formation are the hallmarks spreading beyond the sinuses, destroying surrounding tissues, which may be visible in the nasal mucosa, palate, or skin overlying the orbit [6]. Typically, cerebral mucormycosis occurs as a result of an infection spreading from the sinuses to the eyes and brain. Instances of isolated cerebral mucormycosis are exceedingly uncommon. Disseminated fungal infections are more prevalent among individuals who abuse intravenous drugs, with the basal ganglia being the most frequently affected site in such cases. This is intriguing how the basal ganglia appear to have a preference. When there are elevated levels of iron in this area, it encourages the growth of fungus even more [7]. Timely identification, immediate initiation of medical treatment, and surgical intervention are crucial for effectively managing this life-threatening infection.

Here we report a case of rhino-cerebral mucormycosis triggered after coronavirus disease 2019 (COVID-19) in a diabetic patient who was admitted with acute kidney injury and sepsis. The patient developed periorbital edema and a gradually deteriorating consciousness level, hence the patient was evaluated for mucormycosis.

## Case presentation

A 68-year-old lady, known to have stage 4 chronic kidney disease with diabetes mellitus, essential hypertension, and ischemic heart disease with a history of lymphoma three years back (had six cycles of chemotherapy and 12 cycles of radiotherapy) presented with persistent frontal headache for three weeks. Before this presentation, she was hospitalized one month ago for COVID-19 pneumonia and received steroid treatment but no interleukin (IL)-6 inhibitors. She had recovered from COVID-19 without any complications. Following the headache, she gradually developed an altered consciousness level and peri-orbital swelling (more in the left eye). She experienced low-grade fever, reaching a peak temperature of 101ºF, along with sporadic episodes of vomiting. She did not have any nasal discharge, limb weakness, seizure, or any other systemic complaint.

On presentation, her Glasgow Coma Scale was 10/15; motor, sensory functions, and cranial nerves could not be evaluated properly due to altered consciousness. There was no ophthalmoplegia. The planter was flexor bilaterally. Her pulse was 84/min (normal rhythm and volume), blood pressure 120/80 mmHg, oxygen saturation (SpO2) 96% in room air, and there was no lymphadenopathy. Abdominal examination revealed normal findings and there was no organomegaly. Further assessment with a thorough physical examination revealed no notable findings. There were no apparent signs of ketoacidosis based on clinical assessment.

**Figure 1:**
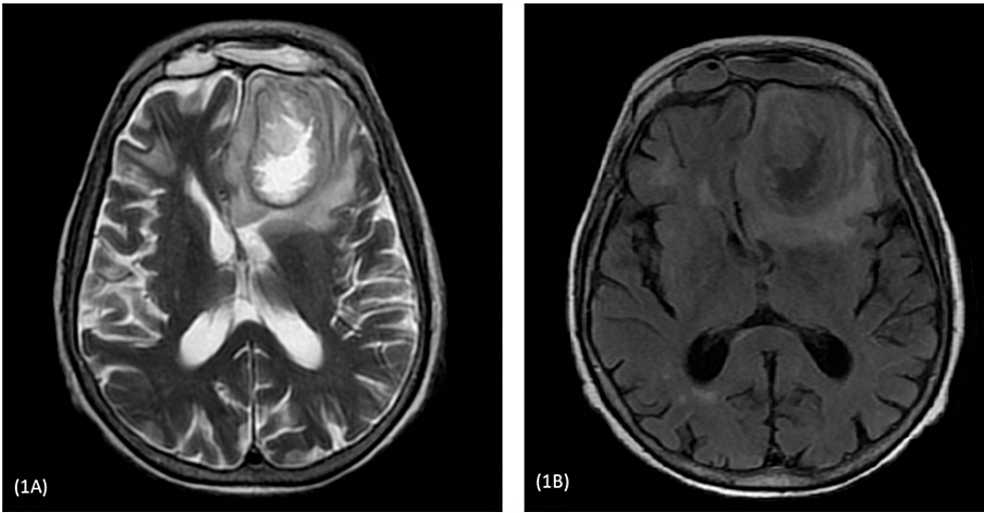
Axial section of T2 weighted image (1A) and T1 weighted image (1B) showing a single large lesion in the left frontal lobe with perilesional edema and central necrosis. Left lateral ventricle mildly compress with rightward midline shifting (9mm).

**Figure 2:**
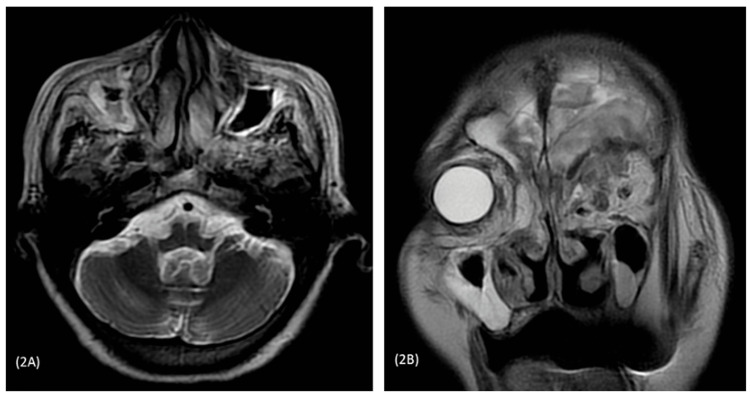
Axial section of T2 image (2A) showing bilateral maxillary sinusitis (right>left); Coronal section (2B) showing infective infiltrate in the left orbit compressing medial rectus muscle.

During admission, her random blood glucose was 10.6mmol/l. Hemoglobin A1c (HbA1c) was 6.9%. Computerized Tomography (CT) scan of the brain revealed a subacute infarct in the frontal lobe and bilateral ischemic change in the periventricular region. CT scan was followed by a Magnetic Resonance Imaging (MRI) of the brain, which showed a large irregular single lesion in the left frontal lobe having perilesional edema and central necrosis with extension to the left orbit. The left lateral ventricle was mildly compressed with rightward midline shifting. Infiltrates were seen along the medial and superior wall of the left orbit which pushed the medial rectus muscle (Figures 1, 2).

The provisional diagnosis was rhino-cerebral mucormycosis. Differential diagnoses included central nervous system (CNS) lymphoma, brain abscess, and cerebral neoplasm.

**Figure 3:**
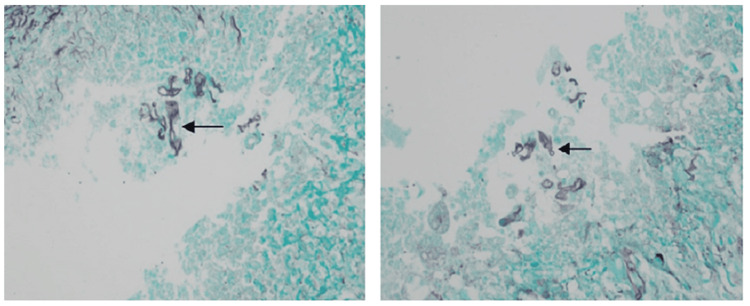
Grocott’s methenamine silver stain (GMS) showing fungal hyphae with granulomatous change.

**Figure 4:**
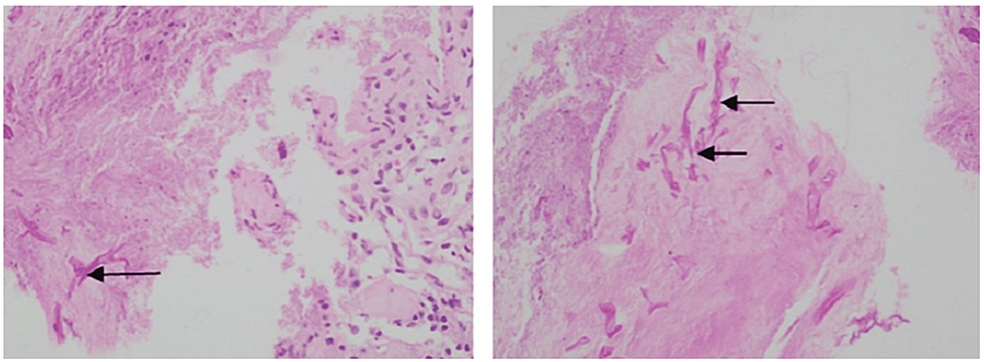
Haematoxilin and eosin stain (H&E) showing broad aseptate fungal hyphae branching at a right angle in the necrosed tissue with granulomatous changes.

Her investigations revealed hemoglobin 10.6 g/dl, total leukocyte count 16.3×10^9^/L; platelet 180×10^9^/L; serum creatinine 2 mg/dl with estimated glomerular filtration rate (eGFR) 4.6 ml/min/1.73m^2^. Her liver function tests showed serum albumin 3.8 g/dl, alanine transaminase (ALT) 26 U/L, and aspartate aminotransferase (AST) 21 U/L, which was within normal limit. She had slightly raised C-reactive protein (42.3 mg/L) and raised serum ferritin level (989 ng/ml). Serial monitoring of absolute lymphocyte count showed a falling trend (Day 1: 8.3%, Day 5: 7.3%, Day 8: 6.6%, Day 10: 5.8%, normal range: 20-40%). A biopsy was performed on the nasal cavity's middle meatus to obtain tissue samples. Histopathological analysis showed a fungal colony of broad aseptate hyphae branching at a right angle in the hematoxylin and eosin stain and Grocott’s methenamine silver stain (GMS) (Figures 3, 4). The subepithelial stroma contained multiple discrete granulomata composed of several foreign body giant cells, eosinophils, and lymphocytes.

Following the histopathological findings of the biopsy report, her treatment was initiated with liposomal amphotericin B at a daily dosage of 5mg/kg. After three days of starting treatment, the patient developed bi-cytopenia (anemia, thrombocytopenia which was managed with blood transfusion), and acute kidney injury (AKI) on chronic kidney disease (CKD) (creatinine rose to 2.5 mg/dl from a baseline of 2 mg/dl). Neurosurgery consultation was taken and conservative treatment was advised because the patient's attendant declined to give consent for surgical debridement considering the age and multiple co-morbidities of the patient. In the later course of the hospital stay (Table 1), the patient developed nosocomial pneumonia with sepsis followed by septic shock.

**Table 1:** Gradual deterioration of biochemical parameters of the patient during the hospital stay ESR: erythrocyte sedimentation rate; CRP: C-reactive protein; PT: prothrombin time; APTT: activated partial thromboplastin time

Date	19.03.22	02.04.22	07.04.22
Hemoglobin	9.8 g/dl	9.1 g/dl	8.3 g/dl
White blood cell count	16.1 K/µl	20.4 K/µl	21.9 K/µl
Platelet	128 K/µl	80 K/µl	51 K/µl
ESR	92 mm in 1^st^ hour	102 mm in 1^st^ hour	125 mm in 1^st^ hour
CRP	42.3 mg/L	87.6 mg/L	134.5 mg/L
Procalcitonin	0.9 ng/ml	1.9 mg/L	2.6 mg/L
Creatinine	1.8 mg/dl	2.2 mg/dl	3.6 mg/dl
PT	13.8 sec	14.6 sec	24 sec
APTT	22 sec	25 sec	38 sec

She also developed a new onset melaena. She was planned for a colonoscopy but her condition deteriorated gradually and she required mechanical ventilation. Later tracheal aspirate showed profuse growth of vancomycin-resistant enterococcus (VRE). After 21 days of hospital stay, her condition further deteriorated and she died after the development of disseminated intravascular coagulation and multiorgan failure as a consequence of severe sepsis.

## Discussion

Mucormycosis is pervasive and can be found in fruits, soil, and decaying vegetation. These fungi can grow rapidly and release a large number of spores that can become airborne [3]. Usually, in most immunologically competent hosts, these spores are eliminated by a body’s phagocytic response. However, in an immunocompromised person, when this response fails, germination will take place as a result of which hyphae will develop. As the fungus spreads, its hyphae make their way into the arteries, where they thrive and multiply within the walls and lumens. This leads to the formation of thrombus in the arteries, restricted blood flow, and ultimately, necrosis of the tissues that may lead to the formation of dry gangrene [3]. It is noteworthy that a meta-analysis of 600 series and 851 cases has identified diabetes mellitus as an independent risk factor for rhino-orbital-cerebral mucormycosis [8]. It tends to alter the body's usual immune response to infections. Uncontrolled diabetes promotes the growth of fungi while reducing chemotaxis and the efficiency of phagocytosis, creating an environment where typically harmless organisms can flourish in an acidic environment. Diabetic individuals, especially those with diabetic ketoacidosis, face an increased susceptibility to mucormycosis resulting from Rhizopus oryzae. These organisms produce the enzyme keto-reductase, enabling them to utilize the patient's ketone bodies [9]. In this case report, the patient was diabetic but there was no feature of diabetic ketoacidosis.

Patients with rhino-cerebral mucormycosis clinically present with malaise, facial pain, and swelling with low-grade fever. Usually, the ailment starts by impacting the nasal mucosa or palate and subsequently extends to the paranasal sinus by invading the neighboring blood vessels, such as the angular, lacrimal, and ethmoidal vessels. Moreover, mucormycosis can also involve retro-orbital region by direct extension [10].

There is some research literature on rhino-cerebral mucormycosis cases associated with COVID-19. In a reported instance of severe COVID-19 accompanied by fungal co-infection, the analysis of cell counts indicated a gradual rise in white blood cell count and neutrophils, while lymphocytes exhibited a progressive decline [11]. It is theorized that the infection caused by severe acute respiratory syndrome coronavirus 2 (SARS-CoV-2) might impact the cluster of differentiation 4 (CD4+) and cytotoxic T cells (CD8+), which play a significant role in the pathological progression of COVID-19. In the COVID-19 cases along with diabetes, due to hyperglycemia, there may be decreased viral response. A delay in the interferon-gamma response along with a prolonged hyperinflammatory state and reduced number of CD4 and CD8 may exacerbate the ‘cytokine storm’ and produce a devastating state of the disease [12].

In a case series, the author reported six cases of rhino-cerebral mucormycosis following COVID-19 from the Indian subcontinent [13]. The average period from COVID-19 diagnosis to the emergence of mucormycosis was recorded as 15.6 ± 9.6 days. Yohai et al. in their meta-analysis reported that the survival rate decreased if there is a delay of more than six days from diagnosis to the start of treatment of mucormycosis [14]. In our case, the patient presented after six weeks of recovery from COVID-19 pneumonia, which may have contributed to the grievous outcome. Careful observation is required in moderate to severe COVID-19, diabetic patients with COVID-19, or those receiving systemic corticosteroids during COVID-19. If these groups of people develop sinus tenderness with nasal discharge, visual disturbance, and ophthalmoplegia, there should be a high index of suspicion for fungal co-infection. These patients should undergo immediate imaging studies. Further, in the absence of clear benefit, the use of glucocorticoid in mild COVID-19 and other drugs targeting immune pathways such as tocilizumab (IL-6 inhibitor) and secukinumab (IL-17 inhibitor) should be discouraged to avoid flaring up of fungal infection.

The optimal approach to treatment involves timely surgical debridement alongside antifungal therapy and addressing the underlying pre-existing condition. Vascular invasion accompanied by thrombosis results in vascular blockage and tissue necrosis, significantly impeding drug penetration and emphasizing the importance of surgical debridement. Combining surgical and medical treatments proves more effective than either approach alone. According to a comprehensive analysis conducted by Roden et al. that involved 929 cases, the success rates for various treatments were documented as 61% (324 out of 532) for cases using amphotericin B, 57% (51 out of 90) for cases undergoing surgery alone, and for cases that underwent both antifungal therapy and surgery, the success rate was 70% (328 out of 470) [15].

The standard medications for treating mucormycosis are amphotericin B preparation and posaconazole. Liposomal amphotericin B is the preferred therapeutic agent due to its favorable side-effect profile when in comparison with amphotericin B [16]. Posaconazole can serve as an alternative treatment option for patients who pose challenges in terms of amphotericin B treatment or exhibit intolerance to it. Nonetheless, it is not advised as the initial treatment choice but can be employed as a secondary medication when transitioning from a previous therapy [17]. Isavuconazole, a newer generation triazole with broad-spectrum efficacy, is utilized in the treatment of mucormycosis. Notably, isavuconazole does not necessitate specific timing or specific food requirements for its administration, distinguishing it from posaconazole.

## Conclusions

In patients with COVID-19 who have underlying medical conditions like diabetes, chronic kidney disease, malignancy, or organ transplantation, it is crucial to maintain a high level of suspicion to consider the possibility of a fungal infection. Failing to promptly diagnose such an infection can lead to serious consequences. We can practice simple bedside examinations like vision, pupil, ocular motility, and sinus tenderness during the evaluation of COVID-19 patients. Immediate treatment initiation is of paramount importance to reduce the mortality rate. We should be more judicious in the use of steroids and other immunosuppressive therapy particularly to avoid flaring up fungal co-infection.
